# Cholera Disarmed—The Triumph of Genius(?)

**Published:** 1892-10

**Authors:** R. B. Leach

**Affiliations:** Paris, Texas


### EDITORIAL DEPARTMENT.

### CHOLERA DISARMED—THE TRIUMPH OF GENIUS(?)

Behold, a new star has arisen; this time in the West,—and *mirabile dictu*, within the bounds of the "malarial belt," which, according to the great Billings, is inimical to talent, and in which genius can never breathe.

Some writer has sagaciously observed that there never was a crisis in human affairs—an emergency to be met, but some genius was raised up to meet it, or words to that effect. The truth of this observation has been demonstrated repeatedly. History, ancient and modern, abounds with striking illustrations of its correctness. Had we not, in the late war, Lee on the one side, and Grant on the other, and Jefferson Davis and Abraham Lincoln, to demonstrate the truth of the observation from the standpoint, respectively, of Yank and Confederate? Have we not in Texas, now, in the present great emergency our own diminutive Demosthenes—a modern Moses—

> "* * tempter sent or tempest tossed—all undaunted,
> On these shores by (imaginary) horrors haunted,"—

to lead a bewildered people out of the labyrinth of a complicated "calamity?" "commission"ed to lead the children of AbraHAM to the promised land, where money will be plenty and fat offices will go a'begging?

So, even, when the "crisis called," *Caius Marius* was on hand. The breach was closed—the country saved,—but Caius M.,—oh, where was he? (We will not follow the *simile* any further, as we are not in politics.)

Well, the cholera came. Arising in the East—like a rested lion he shook his mane and "lit out"—seeking whom he might devour. People fled panic-stricken at his approach, and there was none to stay his progress. Across the Atlantic at a step he came, like a school-boy skipping across the spring branch. He growled at our very door,—and science, palsied with fear, yet with "disinfectants" in hand, stood irresolute.

Behold,—away off in Texas the cry is heard;—the "crisis' having called, the genius was forthcoming!

Dr. R. B. Leach, a highly respectable citizen of Paris, Texas, a homeopathic physician, eminent in his class, and compared to whom, in light of the following, Hahnemann was a chump,—conceives an idea. No, its an inspiration! It is brilliant, thrilling! Hahnemann said, "the hair of the dog will cure the bite," and behold—the homeopathic doctrine—*similia similibus curantur*—is born! This modern Hahnemann now advances the beautiful theory that the hair of the dog will *prevent* the bite, hush the bark and choke off the dog! That "like" will not only cure "like," but will *prevent* it! Moreover, he is a benefactor in another sense. Unlike the Keeleys and the Amicks (sordid creatures) who for sordid gain sell their great discoveries for the ducats,—Dr. Leach hastens to "give it away" without money and without price,—fondly anticipating that time when the world will ring with the echo of his name,—when unborn generations will rise up and call him blessed, and fortune and fame will be his inheritance by right. Does he not intimate as much in his letter? He will be canonized; and embalmed in history his name will go sounding down the ages hyphenated with that of Jenner, and coupled with all that is great and glorious in science.

He has laid his discovery before the *President of these United States*, and begs that it be speedily placed in the hands of the cholera sufferers and the cholera scared people of the world. The time has come! Cholera, like small-pox, must now bow before the power of science, and be disarmed of all its terrors.

This letter to the President Dr. Leach sends to DANIEL'S TEXAS MEDICAL JOURNAL, accompanied by the following courteous request; knowing that we will hasten to give the world the benefit of it, in case Benjamin H. is too busy to attend to it; knowing, too, our appreciation of a *good thing*. Here are the letters:

PARIS, TEXAS, September 29, 1892.

*F. E. Daniel, M. D., Editor Daniel's Texas Medical Journal, Austin, Texas:*

DEAR SIR:—As the exponent of Arsenic vs. Cholera (now in the hands of Health Officer Swearingen*) I enclose you a copy of my expose to the President that you may place it within the reach of all to make personal tests such as are now being made at N. Y. quarantine and the importance of the subject demands. My assertions are pronounced impregnable by the U. S. Marine

*A copy of the letter to the President was sent to State Health Officer Swearingen; we await with breathless anxiety his report on the "tests."

Hospital Bureau, except by such test which I await fearlessly and in confidence; meanwhile courting your esteemed opinion or criticism, or that of any of your readers, I remain, respectfully yours,
R. B. LEACH.

The following is the "expose":

ARSENIC VS. CHOLERA.

PARIS, TEXAS, September 3, 1892.

TO THE PRESIDENT:—Inclosed you will find an introduction to your correspondent, from my friend Gen. S. B. Maxey, which I should have handed you, but my wife's illness keeps me at home for the present. It will give you an inkling of what I hoped to present in person for your honorable consideration had it been possible to go to Washington at this time. Nevertheless I am ready and anxious to defend my theory before the medical fraternity of the world, and hope, should you deem it worthy further consideration, that you present it to them through the channels at your command. I am anxious to substantiate to the satisfaction of the world that Arsenic (pure or attenuated as the *rabies canina* of Pasteur or the cholera *virus* of Haffkine) will prove itself as surely a vaccine against Asiatic cholera, and better and safer than inoculation with cholera *virus*, as are those of Jenner, Pasteur or Haffkine sure antidotes to their respective similars. Arsenic should be affixed in plastic form to ivory "points," in quantity not exceeding 1-30 gr. to the "point," or used in hypodermic injections in doses ranging from two to ten *minims* Fowler's or Pearson's solution.

Arsenic is, with but a few exceptions, destructive to animal and vegetable germ life, and is reconstructive and a tonic to the system, yet in lethal doses produces symptoms similar to Asiatic cholera, the same as does cow pox *virus*, at times, produce like symptoms in man to small pox; as does the *rabies canina* produce symptoms similar to hydrophobia; as does also the cholera *virus* of Haffkine effect similar symptoms as found in epidemic cholera.

Arsenic does all this in a like similarity to epidemic cholera, as does Jenner's cow pox *virus* to small pox. For instance: Let me here quote you from Bartholow, p. 142, where he says: "Arsenic is one of the numerous remedies proposed for the treatment of epidemic cholera;" also from Virchow, who says: "That many cases of arsenical poisoning are not distinguishable by their symptomatology or morbid anatomy from cases of epidemic cholera."

I here call attention of all medical men to the indisputable fact that many patients, before well, vaccinated with cow pox *virus*, have exhibited symptoms so astonishingly like small pox as to question the purity of the *virus*.

I would suggest that all who will acquiesce, especially those in the afflicted districts of Europe, all passengers, officers and sailors, from infected ports, and even cholera patients themselves, especially in the first stage, all suspects or associates of suspects, quarantine officers, their assistants and councilors,* be at once vaccinated with arsenic as above prescribed, or take five-drop doses of Fowler's solution every few hours till slight physiological effect is produced. For the effect of arsenic continues in the system from a few hours to four weeks or longer according to the size and frequency of the dose, and with such as prescribed all may safely feel immunity from attack for at least four weeks, when for safety all still exposed to cholera should be re-subjected to arsenic.

As Koch is under royal favor in his efforts to assist the afflicted, as Pasteur is still recognized as the leader of experimenters in medicine, and Jenner has at last received the plaudits so long due from his colleages and the world,† I have the temerity at this time, to present my theory to the head of our great nation, when all danger signals are flying and all able minded thinkers at thought to devise some method whereby we may possibly be spared the threatened epidemic of a most loathsome and painful and fatal disease, and probably thereby spared a financial panic, the greatest in our history.

With due deference to the opinions of others, but with a firm belief in my own, I present to your honorable attention.

Respectfully submitted, R. B. LEACH.

In presenting this remarkable document to the physicians of Texas, we hardly know whether to venture a criticism or not. The thing is so palpably absurd, looked at from the standpoint of rational medicine, and in the light of modern sanitary knowledge, that it were superflnous to combat any assertions therein contained. The doctor's premises are wrong, and, of course, his deductions are false. It seems that he does not in the least grasp the idea of immunity and infection. The introduction of arsenic into the system cannot be compared to the vaccination with an attenuated virus; the virus contains the living organism of the disease, or its product, and is not an "antidote" to the disease in any sense, but is pathogenic,—it produces the disease, in a much modified form,—and does not *prevent* it, much less *cure* (antidote) it. For the same reason that the person is "immune" after an attack of small pox, is he immune after vaccination; the disease has been introduced into the system by the attenuated virus —and he suffers a mild attack. Who could ever be brought to believe for a moment that, because a person has once experienced the effects of arsenic, he would be insusceptible to a second dose?

---

*[Counselors?—ED.]

†[He's dead now.—ED.]

or that arsenic, because it produces symptoms resembling those of cholera, will prevent the action of the cholera poison— a living, deadly microbe? It is too absurd for serious consideration.

Alcohol, brandy, in sufficient quantity, will produce stupor and heavy breathing, with slow pulse,—a condition of cerebral and pulmonary congestion. Opium, in large doses, will do the same. As well say, put a man fully under the influence of opium and he may drink all he wants and it will not produce stupor, etc., because the opium has forestalled it and has produced those symptoms before the alcohol was imbibed. Or—to say that if a man purge himself with epsom salts, he cannot afterwards have a diarrhœa. Why did not Dr. Leach select tartar emetic instead of arsenic? Tartar emetic will produce like effects.

Besides, there is nothing new about arsenic causing a train of symptoms resembling cholera. It is known that during cholera epidemics evil persons have given arsenic for criminal purposes, supposing it would be thought cholera.

There is one thing we admire about this letter--the writer's assurance. He has much to learn yet, but he does not seem to be aware of it. We recommend him to read Frankel's Bacteriology, or McLaughlin's Physical Theory of Immunity and Contagion.

He assures us that the "test" is being made at the New York quarantine. The profession will wait with much anxiety and impatience for the result. He also says, the U. S. Marine Hospital Bureau surgeons have pronounced his "assertions" impregnable. (That may be, but how about his theory?) It would be interesting to know who of the U. S. Marine Hospital surgeons so pronounced, or who are making the test at the New York quarantine.

***

The cholera scare subsided almost as rapidly as it arose; the disease disappeared. Physicians were at a loss to account for it; but here we have the explanation. The "raging lion" has retired before this new enemy—"arsenic"—realizing the uselessness of measuring strength with such a remedy in the hands of such a genius;—even as Beelzebub cowed and fled before the emblem of the cross.

